# Dynein promotes sustained axonal growth and Schwann cell remodeling early during peripheral nerve regeneration

**DOI:** 10.1371/journal.pgen.1007982

**Published:** 2019-02-19

**Authors:** Melissa Ducommun Priest, Maria F. Navarro, Juliane Bremer, Michael Granato

**Affiliations:** Department of Cell and Developmental Biology, Perelman School of Medicine, University of Pennsylvania, Philadelphia, Pennsylvania, United States of America; Medical College of Wisconsin, UNITED STATES

## Abstract

Following injury, axons of the peripheral nervous system have retained the capacity for regeneration. While it is well established that injury signals require molecular motors for their transport from the injury site to the nucleus, whether kinesin and dynein motors play additional roles in peripheral nerve regeneration is not well understood. Here we use genetic mutants of motor proteins in a zebrafish peripheral nerve regeneration model to visualize and define *in vivo* roles for kinesin and dynein. We find that both kinesin-1 and dynein are required for zebrafish peripheral nerve regeneration. While loss of kinesin-1 reduced the overall robustness of axonal regrowth, loss of dynein dramatically impaired axonal regeneration and also reduced injury-induced Schwann cell remodeling. Chimeras between wild type and dynein mutant embryos demonstrate that dynein function in neurons is sufficient to promote axonal regrowth. Finally, by simultaneously monitoring actin and microtubule dynamics in regenerating axons we find that dynein appears dispensable to initiate axonal regrowth, but is critical to stabilize microtubules, thereby sustaining axonal regeneration. These results reveal two previously unappreciated roles for dynein during peripheral nerve regeneration, initiating injury induced Schwann cell remodeling and stabilizing axonal microtubules to sustain axonal regrowth.

## Author summary

Nerve regeneration requires coordinated responses from multiple cell types after injury. Axons must extend from the neuronal cell body back towards their targets, while surrounding Schwann cells enter a repair cell state in which they promote regeneration. While nerves of the peripheral nervous system can regrow, it is estimated that fewer than 10 percent of patients fully recover function after nerve injury. In order to understand the mechanisms by which peripheral nerves regrow, we used live cell imaging in the zebrafish to observe the process of nerve regeneration, monitoring axons and Schwann cells simultaneously during this process. Using genetic mutants, we identified a role for the molecular motors kinesin-1 and dynein in promoting axonal regrowth. Furthermore, we found that dynein plays an additional role in Schwann cell response to injury. Thus, we demonstrate that molecular motors are required in multiple cell types to promote nerve regeneration.

## Introduction

Axons of the mature peripheral nervous system have retained a remarkable ability for regeneration. Although simple in concept, peripheral nerve regeneration is a complex process that requires extrinsic as well as intrinsic mechanisms. Chief amongst the intracellular mechanisms that contribute to axonal regeneration are microtubule organization and dynamics as well as axonal transport. It has long been known that following injury the pool of dynamic microtubules at the lesion site, as well as axonal transport, increase [1–3]. Given the central role of both microtubule dynamics and axonal transport in promoting axonal regeneration, factors that regulate both processes are prime candidates for regulating peripheral nerve regeneration.

The molecular motor proteins kinesin-1 and dynein are key regulators of both microtubule organization and axonal transport and have both been implicated in peripheral nerve regeneration. Kinesin-1 is an anterograde motor that is essential for maintaining neuronal homeostasis by transporting cargos, including organelles and mRNA, from the cell body toward synaptic terminals. Kinesin-1 has also been shown to drive axonal outgrowth during development and after injury [4,5]. Dynein has similarly been studied for its role in maintaining homeostasis by transporting cargo, however dynein moves cargo retrogradely towards the cell body. Dynein also plays an important role in axonal injury by trafficking injury signals, including components of JNK and ERK MAPK pathways, which are generated at the lesion site and actively transported to the cell body [6,7]. There these injury signals initiate a regenerative response, characterized first by upregulation of regeneration-associated genes that prevent neuronal cell death, and by initiating a genetic program that promotes regrowth of injured axons back to their original targets [8,9].

More recently it has become clear that in addition to its role in retrograde transport, dynein also functions in cytoskeletal organization and maintenance. For example, in *C*. *elegans* dynein regulates local microtubule dynamics in dendrites to promote microtubule stabilization [10]. Additionally, in the axon dynein transports microtubules to establish and maintain microtubule polarity [11–13]. Finally, besides its preeminent role in axonal homeostasis, dynein is also required for Schwann cell development and myelination [14]. Yet despite dynein’s well documented roles in both axons and glial cells, the effects of dynein on the cellular behaviors of regenerating axons and their associated glial cells in intact animals have not been examined.

In order to examine the diverse cellular functions of molecular motors in multiple cell types, we combined genetic mutants with live imaging of nerve regeneration in larval zebrafish, as previously described [15]. This allowed us to study the real-time dynamics of regenerating axons and surrounding Schwann cells in a whole organism context. We find that the molecular motors kinesin-1 and dynein, albeit to different degrees, are both required for axonal regrowth *in vivo*. Furthermore, we find that dynein is also required to initiate injury-induced morphology changes in Schwann cells, however wild type neurons transplanted into otherwise dynein mutant animals are able to regrow robustly, indicating that neuronal dynein is sufficient to promote axonal regrowth. Finally, we find that dynein is dispensable for initiation of axonal regrowth but is required to stabilize microtubules in injured axons to generate persistent, long-range regrowth. These findings elucidate previously unknown roles for dynein in the initiation of injury-induced Schwann cell behaviors, and identify a distinct role for dynein in promoting axonal regeneration through persistent axonal regrowth via microtubule stabilization.

## Results

### Kinesin-1 and dynein are critical for peripheral nerve regeneration *in vivo*

**Fig 1:**
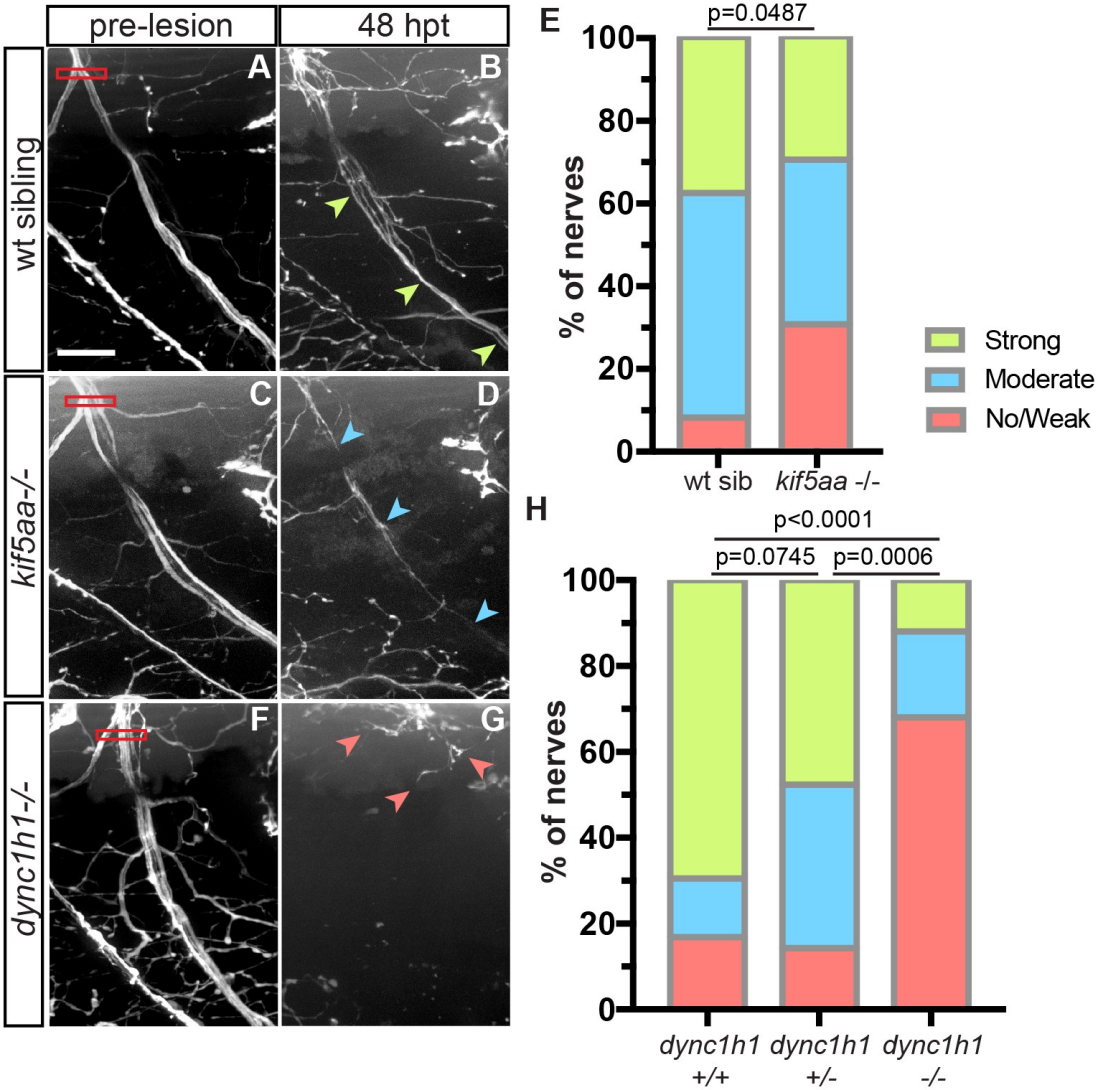
Dynein and Kinesin-1 are required for peripheral nerve regeneration *in vivo*. (A) Wild type motor nerve pre-lesion (red box, transection site; scale bar = 20 μm). (B) By 48 hpt, several fascicles have regrown fully across the ventral myotome (green arrowheads, regrown axons, strong regeneration). (C) *kif5aa*-/- motor nerve pre-lesion. (D) At 48 hpt, some axons have extended across the myotome (blue arrowheads, regrown axons, moderate regeneration). (E) Quantification of *kif5aa* mutant regeneration at 48 hpt (wild type siblings, n = 66 nerves; *kif5aa*-/-, n = 20 nerves, p = 0.0487, Fisher’s exact test). (F) *dync1h1*-/- motor nerve pre-lesion. (G) By 48 hpt, regrowing axons have extended slightly but failed to reach the ventral extend of the myotome (red arrowheads, stalled axons, no/weak regeneration). (H) Quantification of *dync1h1* mutant regeneration at 48 hpt (*dync1h1*+/+, n = 59 nerves; *dync1h1*+/-, n = 21 nerves; *dync1h1*-/-, n = 25 nerves; p = 0.007; p = 0.0006; p<0.0001, respectively, Fisher’s exact test).

To determine the *in vivo* roles of molecular motors in peripheral nerve regeneration, we first assessed regeneration in mutants lacking *kif5aa*, which encodes the neuron-specific Kif5A heavy chain of the conventional anterograde motor Kinesin-1. We have previously shown that laser mediated transection of motor nerves in larval zebrafish initiates a Schwann cell dependent peripheral nerve regeneration program reminiscent of what is observed in adult vertebrates [16]. Following their complete transection at 5 days post-fertilization (dpf), ventral motor nerves exhibit Schwann cell dependent functional regeneration by 48 hours post-transection (hpt) [15] (Fig 1A and 1B). Prior to transection, *kif5aa*-/- motor nerves were indistinguishable from wild type nerves (Fig 1C). By 48 hpt, motor axons in *kif5aa*-/- mutants had regrown across the full extent of the ventral myotome, although when compared to wild type siblings the number of fascicles that reached their ventral targets was reduced (Fig 1D and 1E). Using a previously established semi-quantitative scoring index (for details see Materials and methods and [15] we confirmed that compared to wild type siblings, motor axons in *kif5aa* mutants exhibited reduced regeneration (p = 0.0487, Fisher’s exact test).

We next assessed motor axon regeneration in genetic mutants for the dynein heavy chain gene (*dync1h1*) which encodes a core component of the retrograde motor dynein. Prior to injury at 5 dpf, *dync1h1*-/- motor axons exhibit normal architecture, presumably due to the large maternal load sufficient to promote axonal development [17] (Fig 1F). In contrast, following transection, motor axons in *dync1h1*-/- mutant animals frequently failed to extend beyond the transection site (Fig 1G, quantified in Fig 1H). Analysis of dynein heterozygotes revealed a less severe, although still significant, defect in axonal regrowth, demonstrating a dose-dependent requirement for dynein in promoting axonal regrowth. The severity of the regeneration phenotype we observed in homozygous *dync1h1-/-* mutants was significantly stronger than that present in *kif5aa*-/- mutants. This is consistent with the notion that other heavy chains of Kinesin-1 as well as other Kinesin family motors might compensate for the absence of *kif5aa* [18,19]. In contrast, dynein is the sole protein responsible for microtubule-associated retrograde transport, and therefore the regeneration phenotype we observe in *dync1h1*-/- mutants likely represents a complete block of retrograde transport. We therefore focused on further defining the role of dynein in peripheral nerve regeneration.

### Dynein is required for injury-induced Schwann cell remodeling

In addition to its important and well-studied function in neurons, dynein is also required for proper differentiation and myelination of Schwann cells during development [14]. Furthermore, in zebrafish lacking Schwann cells, regenerating axons sprout from the proximal nerve stump but fail to grow across the injury gap [20], somewhat reminiscent of the phenotype we observe in dynein mutants. Given the importance of Schwann cells for peripheral nerve regeneration and the role of dynein in Schwann cell development, we sought to determine whether dynein is also required for the Schwann cell response to injury, characterized by stereotyped changes in Schwann cell morphology.

**Fig 2:**
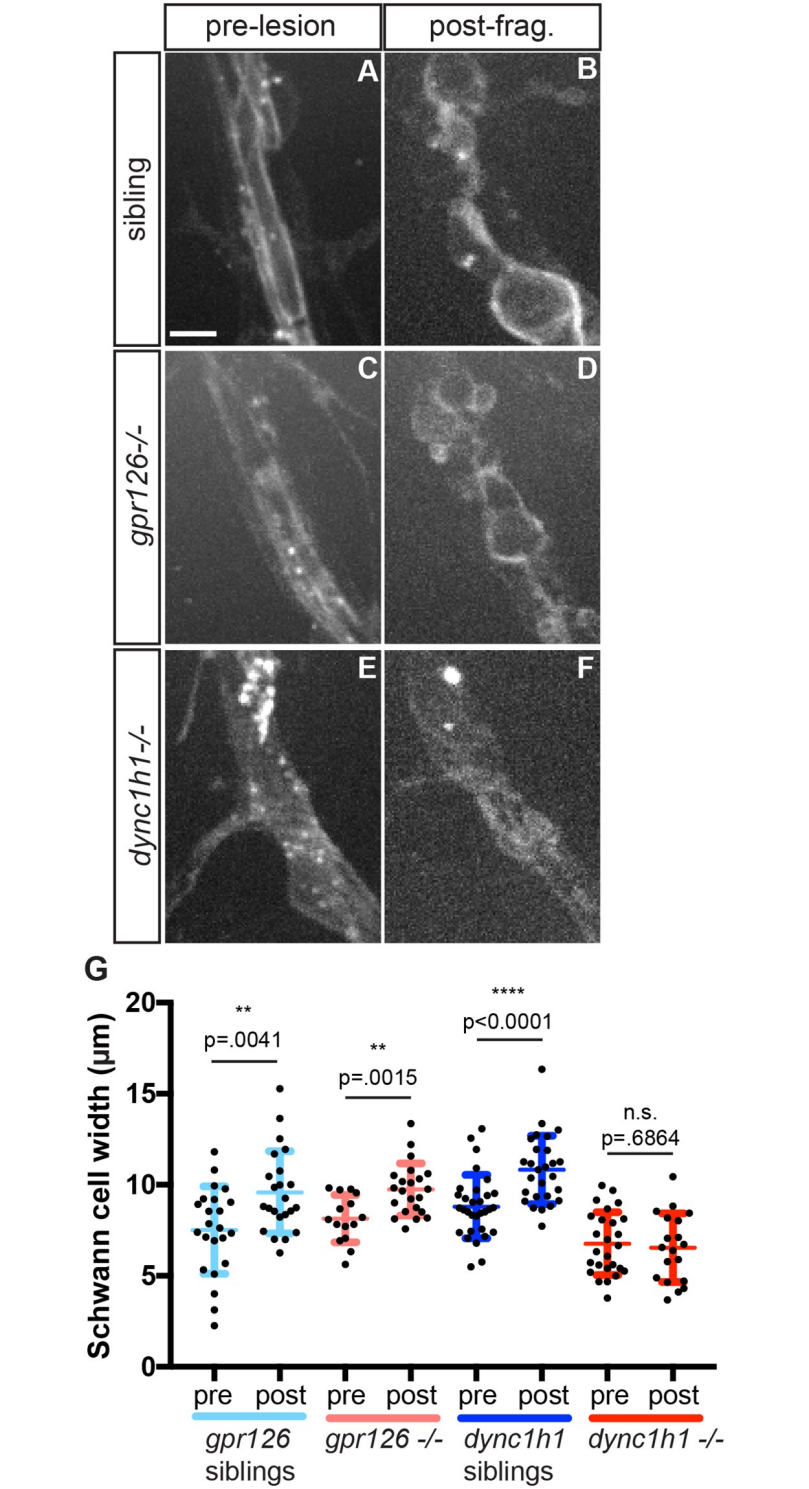
Dynein is required for injury-induced Schwann cell morphology changes. (A-F) Schwann cells in 5 dpf larvae labeled by *Tg*(*sox10*:*mRFP*). (A) Prior to injury, wild type Schwann cells have smooth, straight membranes that are tightly associated with the axonal track (scale bar = 5 μm). (B) After axonal fragmentation, Schwann cell membranes change morphology and widen to accommodate axonal debris. (C) Prior to injury, *gpr126*-/- Schwann cells are loosely associated with axons as they do not myelinate. (D) After axonal fragmentation, *gpr126*-/- Schwann cells are able to change morphology and widen. (E) Prior to injury, *dync1h1*-/- Schwann cells are loosely associated with axons as they also do not myelinate. (F) After axonal fragmentation, *dync1h1*-/- Schwann cell membranes maintain an elongated conformation and do not dramatically change morphology, indicating a disrupted injury response. (G) Quantification of Schwann cell width pre- and post-fragmentation in *gpr126* and *dync1h1* mutants.

We have previously shown that before injury, Schwann cell membranes ensheathe individual motor axons, and that following nerve transection when axons fragment, Schwann cell membranes reorganize, changing from a smooth, tube-like appearance to a more rounded and granular morphology [20], indicative of their transition to an activated, dedifferentiated state, known as the repair cell state that promotes axonal regeneration. Previous studies revealed that in dynein mutants, Schwann cells developmentally arrest at the promyelinating stage [14]. We therefore first wanted to determine whether immature Schwann cells are able to respond appropriately to injury. For this we examined a mutant for the G-protein coupled receptor GPR126, in which Schwann cells also arrest at the promyelinating stage [21], similar to what has been reported for *dync1h1* mutants. Importantly, in contrast to dynein mutants, *gpr126* mutants do not exhibit an obvious deficit in axonal regeneration (n = 37 nerves from 12 +/+ or +/- wild type larvae and 33 nerves from 11 *gpr126-/-* mutants, respectively; p>0.85, Fisher’s exact test). Analysis of Schwann cells dynamics in *gpr126* mutants revealed that Schwann cells respond to injury by extending their membranes dramatically compared to their pre-injury state, indistinguishable from wild type Schwann cells (Fig 2A–2D). This demonstrates that developmentally arrested Schwann cells are still able to respond appropriately to nerve injury.

Having determined that promyelinating Schwann cells are competent to respond appropriately to nerve injury, we next examined the behavior of *dync1h1*-/- mutant Schwann cells. Unlike wild type and *gpr126* mutant Schwann cells, we find that following nerve transection *dync1h1*-/- mutant Schwann cells fail to initiate any morphological changes, and instead retain their pre-injury morphology and membrane position for the duration of the imaging period (up to five hours), arguing against a delay in onset but rather for a complete lack of Schwann cell injury response (Fig 2E and 2F). To quantify this phenotype, we measured the changes in Schwann cell width following nerve transection as a simpler proxy for the complex changes in Schwann cell morphology (Fig 2G). This revealed that while wild type and *gpr126*-/- Schwann cells significantly increase in width after injury, *dync1h1-/-* Schwann cells show no significant change. Thus, while *dync1h1*-/- mutant axons initiate fragmentation following injury, their associated Schwann cells fail to respond, consistent with the idea that dynein is critical for injury-induced Schwann cell remodeling.

### Neuronal dynein is sufficient to promote axonal regrowth

**Fig 3:**
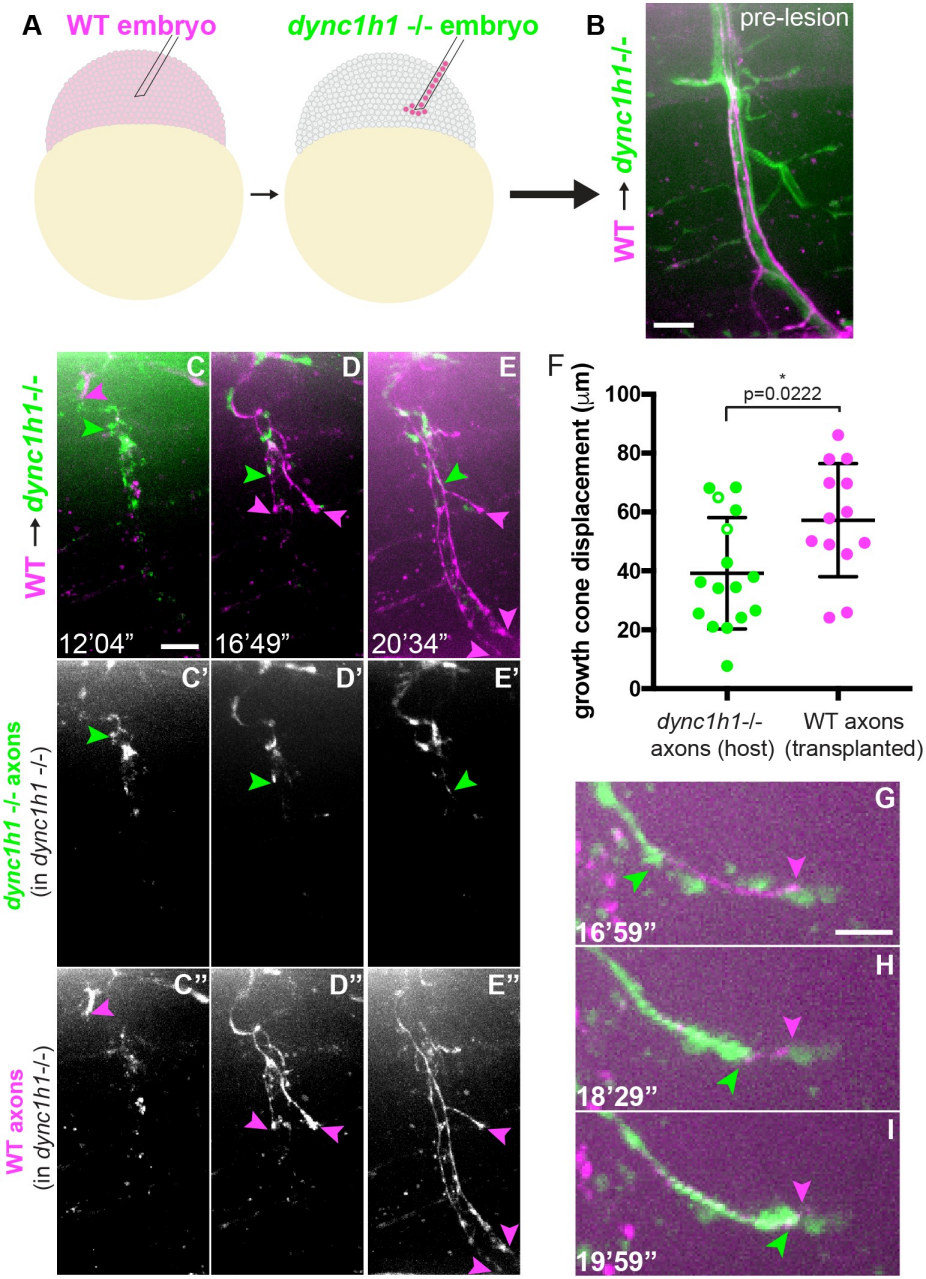
Neuronal dynein is sufficient to promote axonal regrowth. (A) ~10 rhodamine-labeled cells were transplanted from wild type blastulas to *dync1h1*-/- blastulas. (B) At 5 dpf, nerves contained wild type neurons (transplanted cells labeled by rhodamine-dextran, magenta) in a *dync1h1*-/- larva (host motor neurons labeled by *Tg*(*mnx1*:*GFP*), green; scale bar = 10 μm). (C-E) After transection, wild type axons (magenta arrowheads) are able to regrow robustly in the *dync1h1*-/- embryo, while *dync1h1*-/- host axons regrow significantly less (green arrowheads; scale bar = 10 μm). (F) Quantification of growth cone displacement in *dync1h1*-/- host axons and transplanted wild type axons. Open circles indicate *dync1h1*-/- mutant axons that grew along transplanted wild type axons. (G-I) Some *dync1h1*-/- axons demonstrated improved regeneration in the presence of wild type axons in the same nerve. Here, a *dync1h1*-/- axon (green arrowheads) follows along a previously regrown wild type axon (magenta arrowheads; scale bar = 5 μm).

Our results reveal injury-induced phenotypes in two cell types after injury in dynein mutants, and we therefore wondered whether dynein functions in neurons or Schwann cells to promote axonal regrowth. To determine the cell type in which dynein functions to promote axonal regrowth, we generated chimeras at the blastula stage [22] that contained wild type motor neurons and axons in otherwise *dync1h1*-/- larvae (Fig 3A and 3B). Control transplantations have previously shown that wild type cells transplanted into wild type embryos generate motor neurons that are morphologically and functionally unaffected by transplantation [23]. Following development and subsequent transection in a *dync1h1*-/- environment, wild type axons were able to regenerate robustly for the first 9 hours after sprouting (Fig 3C–3F), in a manner indistinguishable from wild type axons in a fully wild type environment. This indicates that restoring dynein specifically in neurons in a dynein mutant is sufficient to promote axonal regrowth, demonstrating a neuron-intrinsic role for dynein during peripheral nerve regeneration.

Interestingly, we found that *dync1h1*-/- axons that had wild type axons in the same nerve regrew more robustly than *dync1h1*-/- axons in nerves with no transplanted cells (14.23 ± 2.06 μm growth in *dync1h1-/-* larvae without transplants, see below; 39.33 ± 4.72 μm growth in *dync1h1-/-* larvae with transplants, Fig 3F). In several instances, we observed *dync1h1*-/- axons growing along previously extended wild type axons (Fig 3G–3I). This indicates that the presence of wild type axonal regrowth is able to partially rescue the *dync1h1*-/- axonal regrowth defects, likely through cell-cell adhesions between the dynein mutant axon and wild type axons.

### Dynein stabilizes axonal growth during regeneration

**Fig 4:**
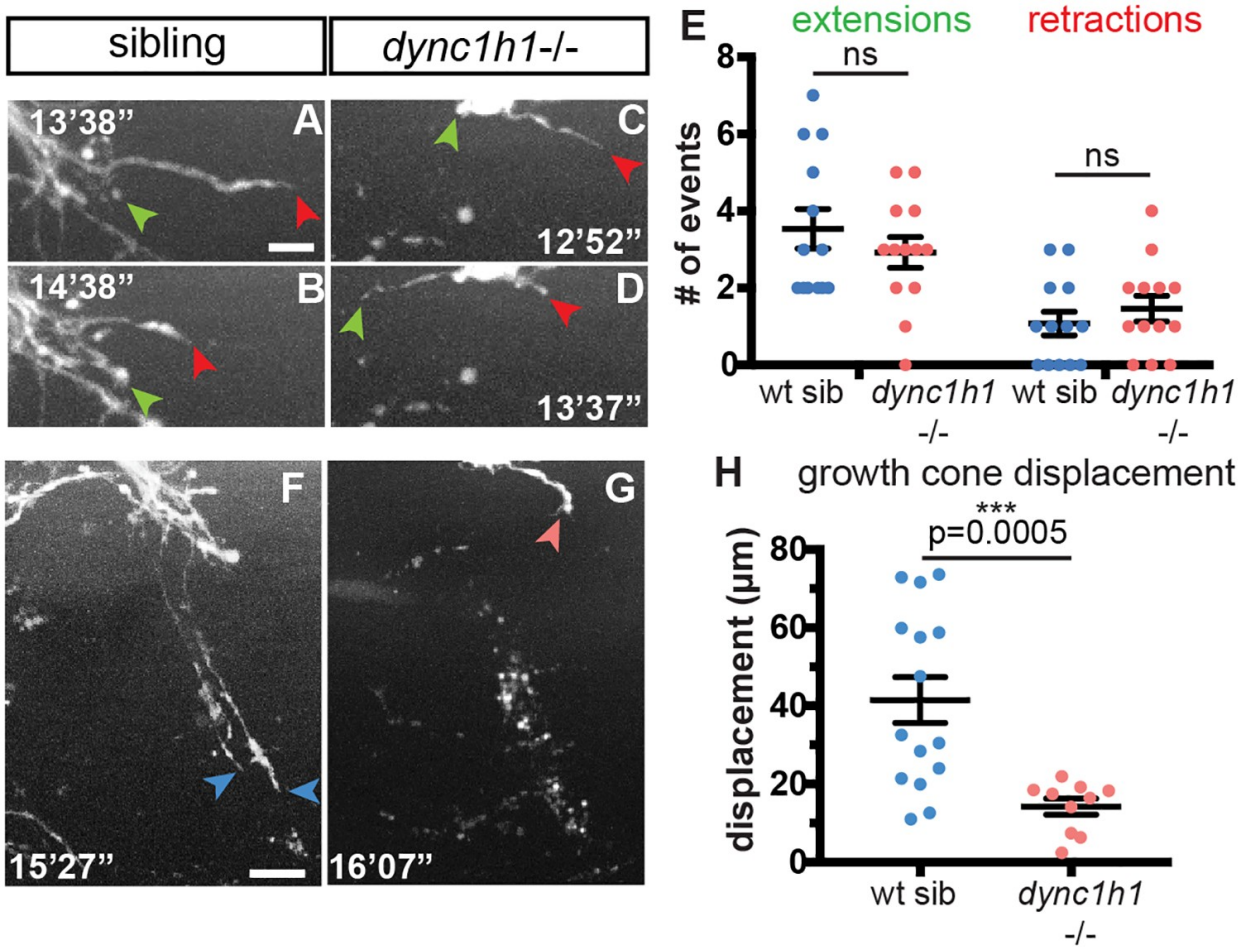
Dynein stabilizes axonal extensions during regeneration. (A-B) In wild type animals, regenerating axons begin probing the environment by extending and retracting (green and red arrowheads, respectively; scale bar = 5 μm). (C-D) *dync1h1*-/- axons also extend and retract after injury. (E) Quantification of extension and retraction events in wild type siblings (n = 13 axons) and *dync1h1-/-* axons (n = 13 axons). (F-G) Measurement of overall growth cone displacement from transection site ~16 hpt in wild type siblings (F; blue arrowheads, growth cones; scale bar = 10 μm) and *dync1h1*-/- (G; red arrowheads, growth cones). (H) Quantification of growth cone displacement ~16 hpt (wild type siblings, n = 15; *dync1h1*-/-, n = 10; p = 0.0005, unpaired t-test).

We next asked how dynein promotes axonal regeneration within peripheral nerves. Peripheral nerve regeneration is a dynamic process composed of several defined stages, starting with growth cones emerging from the proximal stump and starting to probe the injury gap environment. This is followed by stabilization of axonal regrowth across the injury gap and along the correct trajectory, and finally rapid, sustained axonal regrowth towards their original targets [24]. We used live cell imaging after nerve transection to quantify axonal dynamics in dynein mutants and determine which of these stages require dynein. In wild type siblings, we observed growth cones emerging from the proximal stump extending (3.54 events per 8 hours) and retracting (1.08 events per 8 hours) repeatedly, consistent with the idea that these growth cones are probing the injury gap for a path towards their original targets (Fig 4A and 4B). We found that *dync1h1*-/- axons exhibit similar frequencies of axonal extensions and retractions (Fig 4C and 4D), suggesting that they probe the injury gap as actively as their wild type siblings (Fig 4E).

We next examined the second stage of axonal regeneration when axons become stabilized and then extend toward their original targets. To quantify this process we measured the overall displacement of regenerating growth cones over the first ~8 hours after sprouting began. We found that the majority of regenerating wild type axons grew beyond the transection site within 8 hours of sprouting (Fig 4F), travelling an average of 41.49 μm (SEM ± 5.84) over this time period. In contrast, regenerating *dync1h1*-/- axons rarely extended beyond the transection site (Fig 4G), travelling an average of 14.23 μm (SEM ± 2.06) and never exceeding 21.94 μm in growth. Moreover, quantification of growth cone displacement at 8 hours post transection revealed that compared to regenerating wild type axons, *dync1h1*-/- axons exhibited a significant decrease in axonal extension (Fig 4H). Combined these results argue that rather than initiating growth cone sprouting and short range axonal extensions, dynein predominantly acts early during axonal regeneration to stabilize regenerating axons thereby promoting persistent, long-range regrowth.

### Dynein stabilizes microtubules to promote persistent regrowth

**Fig 5:**
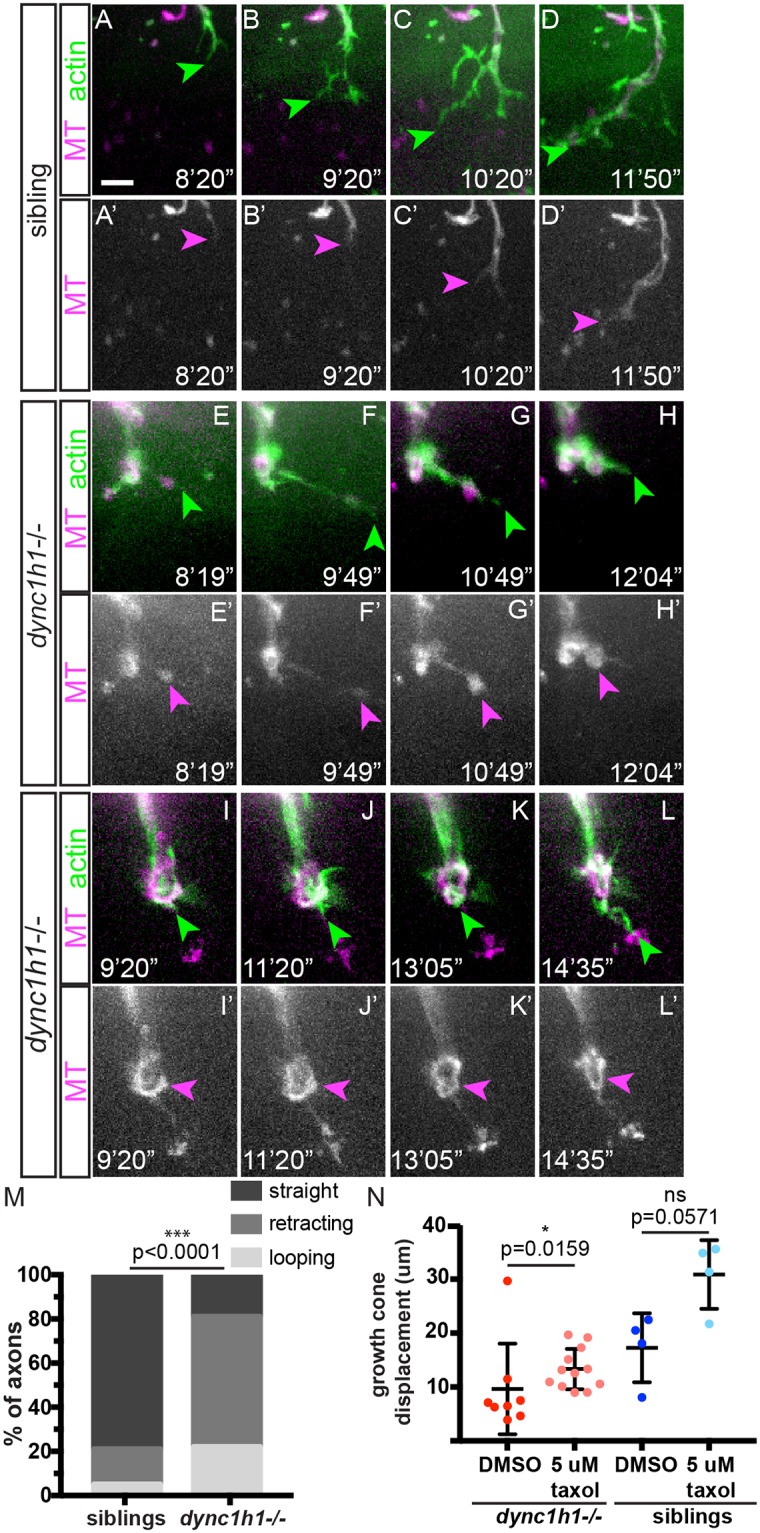
Dynein stabilizes microtubules to promote persistent regrowth. (A-D) Regenerating wild type axons first extend actin protrusions then extended microtubules, leading to stable growth (scale bar = 5 μm; green arrowheads, actin; magenta arrowheads, microtubules). (E-H) *dync1h1*-/- axons extend actin protrusions followed by microtubule growth that arrests during growth cone engorgement and leads to axon retraction (G,H). (I-L) *dync1h1*-/- axons extend actin protrusions but microtubules form aberrant loop structures (magenta arrowheads), preventing further regrowth. (M) Quantification of microtubule organization in regrowing axons of *dync1h1* mutants (siblings, n = 19 axons; *dync1h1*-/-, n = 37 axons; p<0.0001, Fisher’s exact test). (N) Quantification of growth cone displacement ~12 hpt with and without taxol treatment (*dync1h1-/-* with DMSO, n = 8, *dync1h1-/-* with 5 μM taxol, n = 12, p = 0.0159; wild type siblings with DMSO, n = 4, wild type siblings with 5 μM taxol, n = 4, p = 0.0571).

Dynein has recently been shown to play a critical role in generating and maintaining microtubule organization, both processes central to axonal growth [10,11,25,26]. To determine whether dynein regulates microtubule dynamics in axons during regeneration, we used a transgenic line that simultaneously labels actin and microtubules in motor neurons (*mnx1*:*Gal4*; *UAS*:*lifeact-GFP-v2a-EB3-RFP*). Growth cone extension occurs in three stages: first, protrusion driven by F-actin, then engorgement driven by microtubule-based transport of organelles and vesicles, and finally consolidation in which the growth cone contracts and stabilizes to form a cylindrical axon shaft [27]. In regenerating wild type axons, filopodia extend at the growth cone and microtubules follow behind, stabilizing and consolidating newly formed protrusions (Fig 5A–5D). The majority of regenerating *dync1h1*-/- axons (n = 30/37) displayed one of two phenotypes characteristic for microtubule disruption. In 59 percent (n = 22/37) we observed filopodia extension followed briefly by microtubule extension (Fig 5E and 5F) and then arrest at the engorgement stage before finally retracting (Fig 5G and 5H). In 22 percent (n = 8/37) of regenerating *dync1h1*-/- axons, microtubules faithfully followed filopodia extending at growth cones. However, rather than consolidating in the proximal growth cone, they adopted a looped conformation at the leading edge of the growth cone, leading to stalling and retraction (Fig 5I and 5M). The remaining 19 percent had straight, ordered microtubules (n = 7/37). This suggests that a lack of dynein may lead to loss of microtubule organization at regenerating growth cones and stalling of regenerating axons early during the regeneration process. To determine whether stabilizing microtubules during axonal regrowth could compensate for a lack of dynein, we transected nerves in dynein mutant larvae and subsequently treated the larvae with taxol. We used timelapse imaging to assess axonal regrowth dynamics and found that in dynein mutant embryos stabilizing microtubules with 5 μm taxol partially rescued axonal regrowth (Fig 5N). Combined, these findings support a model by which dynein plays a critical role in regulating microtubule dynamics, thereby stabilizing growth of regenerating axons as they initiate their trajectory across the injury gap and towards their original targets. Thus, we demonstrate a role for dynein in promoting axonal extension via microtubule stabilization, as well as a previously uncharacterized role in initiating Schwann cell response to injury.

## Materials and methods

### Ethics statement

All experiments were conducted according to an Animal Protocol fully approved by the Uni- versity of Pennsylvania Institutional Animal Care and Use Committee (IACUC) on January 24, 2014, protocol number 803446. Veterinary care is under the supervision of the University Laboratory Animal Resources (ULAR) of the University of Pennsylvania.

### Zebrafish genetics and transgenes

All transgenic lines were maintained in the Tübigen or Tupfel long fin genetic background and raised as previously described [22]. The *Tg*(*mnx1*:GFP)^ml2^ line [28] was used to label spinal motor nerves and the *Tg*(*sox10(7*.*2)*:*mRFP*)^vu234^ line [29] was used to label Schwann cells. The *Tg(UAS*:*lifeact-GFP-v2a-EB3-RFP*) line was used to label microtubules and actin. The *dync1h1*^*hi3684Tg*^ [30] and *gpr126*^*stl47*^ [31] mutant strains were used and genotyping protocols were performed as previously described.

### Nerve transection and live imaging

Nerve transection and live imaging were performed as previously described [15].

### Axon growth extent quantification

Axon growth extent quantification was performed as previously described [20]. Transected nerves in which axons failed to regrow or did not extend through the entire length of the ventral myotome are categorized as “no/weak regeneration.” Nerves with at least one fascicle that extended through the entire length of the ventral myo- tome are categorized as “moderate regeneration.” Finally, nerves with two or more fascicles extending through the entire length of the ventral myotome are categorized as “strong regeneration.”

### Axon extension and retraction quantification

Axons were imaged every 15 minutes from ~7 to ~16 hpt. Extensions and retractions were defined as growth or retraction of >1 μm between timelapse frames and number of extension and retraction events was counted. Continued movements of the same direction in a subsequent frame were not counted as new events. Measurements were performed on each visibly distinct axon in a nerve.

### Growth cone displacement quantification

Axons imaged at ~16 hpt were measured by drawing a line from the spinal cord exit point to the growth cone. Measurements were performed on each visibly distinct axon in a nerve.

### Schwann cell width quantification

Axons and Schwann cells were imaged before transection and every 15 minutes from ~1 to ~5 hpt. Schwann cell width was measured at the widest point in pre- and post-transection images. Using ImageJ, a line was drawn from one edge of the Schwann cell membrane to the other in an orientation perpendicular to the motor nerve and was measured in microns.

### Cell transplantation for chimera analysis

Cell transplantations were performed as previously described [32]. Wild type cells were transplanted into *dync1h1-/-* embryos in areas known to develop into motor neurons. Larvae were screened at 5 dpf to identify nerves that contained transplanted motor neurons and no other transplanted cell types along the path of the ventral motor nerve. Transection, imaging, and quantification of growth cone displacement in identified nerves were performed as described above.

### Taxol treatment protocol

Larvae were transected according to the above protocol. 3 hours after transection, embryos were bathed in embryo medium with either 1% DMSO or 1% DMSO and 5 μM taxol (paclitaxel, Life Technologies #P3456). When mounted for overnight timelapse imaging, larvae were bathed in Ringer’s solution with either 1% DMSO or 1% DMSO and 5 μM taxol for the duration of imaging.

### Image processing

Image stacks were compressed into maximum intensity projections (MIPs) in Slidebook 6 then processed using ImageJ and Photoshop to normalize brightness and contrast.

### Statistical analysis

Fisher’s exact and Student’s t tests were performed on all applicable datasets.

## Discussion

### Dynein stabilizes axonal extensions during regeneration

Nerve injury induces a local signaling cascade that leads to the production of axon intrinsic signals at the lesion site [33]. There is overwhelming evidence that dynein is critical to transport these injury signals from the lesion site to the cell body where they initiate a neuronal injury response [34–36]. We find that in presumptive dynein null mutants, injured neurons robustly respond to the injury and within ~8–10 hours, regenerating axons sprout from the proximal stump, indistinguishable from what we observe in wild type animals. This raises the question whether axonal sprouting can occur independently of dynein-dependent injury induced signals, or whether in our zebrafish model dynein-mediated retrograde transport is less important to mount an injury response? One clear difference between rodent models and our model is the distance between the injury site and the neuronal cell bodies. In rodent sciatic nerve models lesions are introduced millimeters away from neuronal cell bodies [36], while in larval zebrafish—due to the smaller animal size—lesions are generated about 10–50 μm away from neuronal cell bodies [15] Thus, it is conceivable that due to the almost 100-fold reduction in distance between lesion site and cell body, injury signal propagation from the lesion site to the cell body is less dependent on dynein function. Although it remains unclear how injury signal propagation can occur independent of dynein, this provided us with the unique opportunity to examine dynein’s role in peripheral nerve regeneration beyond its role in injury signal transport.

Endpoint analyses at 48 hpt uncovered a clear role for dynein in peripheral nerve regeneration, with clear effects on both axonal regrowth as well as on injury-induced Schwann cell remodeling (Figs 1, 2 and 4). Using live-imaging to visualize the early stages of the regeneration process, we found that dynein promotes the stabilization and growth of long-range axonal projections, providing compelling evidence that apart from its well-documented role in retrograde injury signal transport, dynein also plays a critical role in sustaining axonal regrowth. Moreover, simultaneously visualizing the cellular behavior of both axons and Schwann cells revealed that loss of dynein prevented injury-induced Schwann cell remodeling. The transition of Schwann cells from their fully differentiated state to a repair cell state is a well-documented and integral aspect of peripheral nerve regeneration [37,38], accompanied by dramatic morphological changes to the Schwann cell, as the cell breaks down its myelin and extends its membrane to engulf axonal debris [39,40]. Dynein regulates several steps of membrane trafficking, including ER to golgi transport, as well as endosomal trafficking [41], so it is conceivable that dynein plays a direct, cell-autonomous role in this process. Alternatively, the inability of Schwann cells to initiate the remodeling process might be a consequence of strongly reduced axonal regrowth, and future experiments will be required to test a possible Schwann cell-specific role for dynein in the remodeling process.

Given that dynein mutants exhibit defects in axonal regrowth and Schwann cell morphology, we performed chimeric analysis experiments. These experiments revealed that dynein function in injured neurons is sufficient to sustain axonal regeneration. Importantly in our chimera experiments, of the roughly 60 axons contributing to an individual motor nerve [42], on average only 1–3 transplanted wild type axons were present. This low level of chimerism was critical to evaluate regrowth capacity of individual wild type axons. This also revealed that the presence of individual wild type axonal regrowth facilitated regrowth of individual, neighboring dynein deficient axons (Fig 5G–5I). At the same time, the low level of chimerism precluded us from asking whether neuronal dynein restored all aspects of peripheral nerve regeneration, including the overall robustness of axonal regrowth for a whole nerve and injury-induced Schwann cell remodeling. Thus, while neuronal dynein plays a critical role in sustaining axonal regrowth, we cannot exclude the possibility that dynein function in Schwann cells also contributes to peripheral nerve regeneration.

### Dynein promotes axonal regeneration by modulating microtubule dynamics

Cytoskeletal dynamics are critical to growth cone formation [43], axonal outgrowth during development [44], and axonal regeneration [45]. Previous studies have revealed that microtubule stabilization promotes axonal regrowth after injury both *in vitro* and *in vivo* [46–48]. Interestingly, studies of *C*. *elegans* dynein heavy chain mutants recently revealed that dynein acts locally in dendrites to stabilize microtubules [10]. This raised the possibility that dynein may also act locally in regenerating axons to stabilize microtubules. We assessed cytoskeletal dynamics during regeneration using a transgene that allowed us to visualize actin and microtubules simultaneously in live, regenerating axons. This revealed that while actin dynamics were grossly unaffected in dynein mutant axons, microtubules often appeared unstable and disordered, with some axons exhibiting looping microtubule configurations reminiscent of those seen in the dendrites of *C*. *elegans* dynein heavy chain mutants [10]. Thus, our results provide compelling evidence that besides its well-documented role in retrograde transport, dynein also promotes microtubule stability critical for growth cone advancement [49], providing a potential mechanism for the rapid and sustained extension observed during wild type axonal regrowth, and deficient in dynein mutants (Fig 2).

Dynein is also known to modulate microtubule dynamics is through microtubule sliding [50], providing an alternative mechanism. This might be a direct effect or may affect microtubule sliding indirectly via modulation of kinesin-1 as these motors have been shown to transport each other directly with one another [51]. Taken together, our results suggest that beyond its function in retrograde injury signaling dynein has a multifaceted role in nerve regeneration that warrants further studies.

## Supporting information

S1 Movie Wild type axon regrowth in a dynein mutant background. (MP4)

S2 Movie Wild type axon regrowth dynamics. (MP4)

S3 Movie Dynein mutant axon regrowth dynamics. (MP4)

S4 Movie Wild type cytoskeletal dynamics during axonal regrowth. (MP4)

S5 Movie Dynein mutant cytoskeletal dynamics during axonal regrowth (stalling). (MP4)

S6 Movie Dynein mutant cytoskeletal dynamics during axonal regrowth (looping). (MP4)
